# Deep Learning-Augmented Zero Echo Time MRI Increases Diagnostic Confidence for Osseous Assessment in Hand and Foot MRI Protocols

**DOI:** 10.3390/diagnostics16152363

**Published:** 2026-07-27

**Authors:** Carina Obermüller, Karolina Pawlus, Maelene Lohezic, Jose de Arcos Rodriguez, Roman Guggenberger, Malwina Kaniewska

**Affiliations:** 1 Department of Diagnostic and Interventional Radiology, University Hospital Zurich, 8091 Zurich, Switzerland; 2 GE HealthCare, 8152 Glattbrugg, Switzerland; 3 GE HealthCare, 28023 Madrid, Spain; 4 Klinik für Radiologie und Nuklearmedizin, Kantonsspital Winterthur, 8400 Winterthur, Switzerland

**Keywords:** magnetic resonance imaging, joint diseases, hands, ZTE, deep learning

## Abstract

**Background/Objectives:** This study assesses the outcomes of integrating deep learning-augmented zero echo time (ZTE DL) MRI sequences into standard MRI protocols for assessment of the hands and feet. **Methods:** In this single-center, retrospective study, a standard MRI protocol of hands and feet at 1.5 T was compared with the same protocol with an added ZTE DL sequence. Pathological changes in the bone, including subcortical sclerosis, osteophytes, joint space narrowing and fractures, were rated as present or absent. Indeterminate intraosseous lesions (erosions/ganglia) and indeterminate extraosseous lesions (ossicles/soft tissue calcifications) were additionally assessed on a semi-quantitative 4-point Likert scale. Diagnostic confidence was rated as low, moderate, or high. Standard MRI vs. ZTE-DL-augmented MRI comparisons were evaluated using a mixed-effects model with reader as a random effect, and X-ray vs. ZTE-DL-augmented MRI comparisons using a paired Wilcoxon signed-rank test. Interreader agreement (two readers) was assessed using Kappa statistics. **Results:** The cohort encompassed 59 datasets (feet = 22, hands = 37) of 40 patients with an average age of 51.79 (SD ± 11.84) years (58% women (*n* = 34), 42% men (*n* = 25)). Additional ZTE DL sequences resulted in similar findings, but with higher diagnostic confidence for assessment of bone changes when compared to conventional MR sequences (*p*-values < 0.05) and, overall, when compared to radiographs in a subgroup (*p* < 0.05 for five of six pathologic bone changes). Interreader agreement of diagnostic confidence was moderate to substantial (kappa 0.59–0.71). **Conclusions:** Addition of ZTE DL sequences to standard MRI protocols of hands and feet at 1.5 T demonstrated similar findings in the assessment of pathological bone changes, but with higher diagnostic confidence compared to conventional MR sequences and radiographs. However, further validation against a reference standard is required to determine diagnostic accuracy.

## 1. Introduction

Radiographs are the first step in the evaluation of the hands and feet [1,2]. However, for specific pathologies such as rheumatoid arthritis or early inflammatory changes, other modalities including ultrasound or magnetic resonance (MR) imaging may be performed, especially for evaluating soft tissue changes [3]. Bone changes, on the other hand, traditionally remain best evaluated on modalities involving ionizing radiation such as X-rays and CT and may be difficult to evaluate on conventional MR imaging, especially in the small joints of hands and feet.

In conventional MRI protocols, pathologic bone changes are primarily assessed on conventional T1-weighted 2D images [4] and are usually detected indirectly by suppression of bright fatty bone marrow signals. However, T1-weighted images lack the 3D visualization of techniques such as CT and the suppression of non-mineralized structures [5], which both potentially obscure detection of small bony changes. Recent developments, including advanced MRI sequences such as Ultrashort Echo Time (UTE) and Zero Echo Time (ZTE) sequences, focus on improving the ability of MRI to detect subtle pathological changes in bone structure [5]. UTE and ZTE MR imaging sequences are based on selective visualization of structures with short T2 * relaxation times, such as bone, tendons, and ligaments. Conventional sequences are limited by these short T2 * relaxation times, in which their signal decays completely before readout, resulting in signal voids. UTE/ZTE sequences overcome this by utilizing near-instantaneous data acquisition to capture these rapidly decaying signals. As with CT, UTE/ZTE sequences provide true three-dimensional imaging, allowing for high-quality multiplanar reconstructions and assessment of cortical and trabecular bone. UTE/ZTE sequences have shown the potential to mimic CT imaging in several body regions, allowing for improved visualization of subtle bone abnormalities, for example the skull bone [6], the shoulder [7], the knee [8,9], the lumbar spine [10], the osseous craniocervical junction [11] and the osseous cervical spine [12,13], and in medication-related osteonecrosis of the jaw [14].

Additionally, previous research has demonstrated the potential of deep learning (DL) based reconstruction techniques in accelerating MRI acquisition and improving image quality, especially for shoulder, knee and lumbar spine [7,9,10,15,16,17,18]. Other approaches to decrease acquisition times, such as parallel imaging or compressed sensing [19,20,21], are now being replaced or enhanced by DL reconstruction algorithms [22,23]. These advancements in DL reconstruction algorithms have the potential to reduce scan times while maintaining diagnostic accuracy and reader confidence [18,24]. In the context of ZTE MR, DL augmented ZTE (ZTE DL) sequences have shown potential in further enhancing the quality of ZTE MRI images, including the shoulder [7], the pelvis [25] and the knee [8].

Therefore, the inclusion of ZTE DL sequences for imaging of small joints such as hands and feet could provide more comprehensive evaluation of pathologic bone changes without the need for additional imaging involving radiation exposure. This study evaluates the hypothesis that integrating ZTE DL MRI sequences into a conventional MRI protocol will provide a more comprehensive evaluation of pathologic bone changes in hands and feet by increasing diagnostic confidence.

Key points

• Pathologic bone changes in small joints in the hands and feet are traditionally assessed on radiographic exams, while soft tissue changes can be better evaluated using MRI.
• Deep learning-augmented zero echo time (ZTE DL) MRI sequences, which operate with a negligible echo time determined only by transmit–receive recovery time, enable assessment of pathologic bone changes in small joints in the hands and feet at 1.5 T.
• The addition of ZTE DL sequences to a conventional MRI protocol of hands and feet at a 1.5 T MRI scanner showed higher diagnostic confidence for the assessment of pathologic bone changes than with conventional sequences only.

## 2. Methods and Materials

Ethical approval was obtained from the ethics committee prior to this retrospective study. The retrospective cohort encompassed consecutive MRI examinations of hands and feet between July 2022 and December 2023 that were referred to our institution. The indication for these scans included structural changes associated with rheumatoid diseases (*n* = 45), evaluation of changes due to trauma or suspected fracture (*n* = 9), evaluation of suspected infection (*n* = 2) and evaluation of suspected neuroma (*n* = 3). Patient studies were excluded if not all regions listed in Table 1 were included in the field of view, in case of incomplete storage of raw data or missing reconstruction of deep learning-augmented ZTE sequence, and if patient age was below 18 years at the time of the study.

### 2.1. MRI Protocol and MR Acquisition

All MRI examinations were performed using a 1.5 T MRI scanner (SIGNA Artist, GE HealthCare, Waukesha, WI, USA). 3D isotropic radial ZTE pulse sequences were acquired in addition to the conventional MRI protocol of the hands or feet. Detailed scan parameters of the ZTE sequence are shown in Table 2.

In 32 out of the 37 hand scans, a 19-channel head and neck coil was used; in four hand scans, a 16-channel FM coil; and in one hand scan, a 16-channel FL coil was used, as dedicated hand or wrist coils were not available at our institution. Upon review of each case and discussion with senior MSK radiologists, the differences between these coils for the ZTE DL sequence were deemed not significant for this study, and thus both were included in the study cohort. All MRIs of the feet were performed using an 8-channel foot and ankle coil.

Detailed scan parameters of each protocol can be found in the Supplementary Materials in Supplementary Tables S1 and S2. A coil-level sensitivity analysis addressing potential coil variation is provided in Supplementary Table S3.

ZTE sequences were reconstructed in a coronal plane and then transferred to a multiplanar viewer.

### 2.2. Deep Learning Reconstruction of ZTE DL Sequences

The ZTE sequences were acquired with a 3D Archimedean spiral radial k-space trajectory, and the gridded k-space data were automatically reconstructed by a prototype DL-based reconstruction software running on the scanner. These sequences were finally labelled as ZTE DL sequences.

The DL based reconstruction used in this study is an extension of the commercially available AIR™ Recon DL algorithm (GE HealthCare, Waukesha, WI, USA), adapted for 3D ZTE acquisitions. It is based on a deep convolutional neural network (CNN) and enhances MRI images by reducing noise and minimizing artifacts while preserving edge sharpness. It is integrated into the scanner’s system and processes the acquired ZTE k-space data. The CNN of the AIR™ Recon DL algorithm contains 4.4 million parameters across 10,000 kernels and was trained using supervised learning with high-resolution, low-noise images and their lower-quality counterparts [26]. This CNN is integrated into the scanner’s native inline reconstruction pipeline via dedicated post-processing software (Orchestra SDK, GE HealthCare, Waukesha, WI), which applies it to the raw, complex-valued, full bit-depth k-space data, rather than an already-reconstructed magnitude image, to reduce noise and minimize Gibbs ringing/truncation artifacts while preserving edge sharpness.

The ZTE DL prototype reconstruction allows for selection of the noise reduction factor between 0 and 100%. For this study, images were processed with maximum noise reduction at 100% as described in previous studies from our group [7,8,13,18]. The processed ZTE DL volumes were subsequently transferred to and reviewed in the department’s PACS system (DeepUnity Diagn, Dedalus, Bonn, Germany).

### 2.3. Conventional Radiographs

In a subgroup of 22 scans (hands: *n* = 12, feet: *n* = 10), conventional radiographs were available, with a minimum time distance to the MR scan of 2 days, a maximum of 440 days, and a median of 34 days. Radiographs were available in PA/AP projections.

### 2.4. Image Analysis

Readout was performed independently by two radiology residents (CO, KP), with one and three years of experience in MSK radiology, respectively, after a readout of 10 unrelated image sets. The equivocal cases were discussed with senior MSK radiologists until consensus was reached. Both readers were trained in the assessment protocol by an experienced MSK radiologist prior to readout. As ZTE DL sequences are intended for use beyond dedicated MSK radiology practice, this study design intentionally reflects an all-day clinical reading scenario with non-expert readers. Evaluating how reader experience modifies the ZTE DL benefit would require a dedicated study design and is beyond the scope of the present work.

Case order was randomized within each reading session, and both readers were blinded to clinical information; the second reading was performed fully independently of the first. Randomization of case order was considered a sufficient method for counterbalancing the order of reads. Readout was performed for the conventional MRI protocol first, followed by a separate evaluation of the conventional MRI protocol with the addition of ZTE DL sequences after 2 weeks; this interval was considered sufficient to minimize recall bias while remaining practical for the clinical reading workflow. The subset in which conventional radiographs were available was assessed by one reader after 4 weeks.

### 2.5. Degree of Pathologic Bone Changes

Six features were assessed in five specific regions of both hands and feet (Table 1). Subcortical sclerosis, osteophytes, joint space narrowing and fractures were assessed as present or absent. Because distinguishing erosions from intraosseous ganglia and ossicles from soft tissue calcifications was unreliable at 1.5 T, we grouped these findings as combined categories for the readout, hereafter referred to as “indeterminate intraosseous lesions” and “indeterminate extraosseous lesions”, respectively. Hence, indeterminate intraosseous lesions and indeterminate extraosseous lesions were assessed on a semi-quantitative 4-point Likert scale (0 = none, <3 = 1, 3–6 = 2, >6 = 3).

### 2.6. Diagnostic Confidence

Diagnostic confidence was rated for each pathologic bone change on a scale of 1 to 3 (1 = 50/50 chance, not sure; 2 = moderate confidence, diagnosis probable; 3 = sure of diagnosis).

### 2.7. Statistical Analysis

Statistical analysis was performed using Python 3.12.1 with pandas, SciPy 1.13.0, and scikit-learn libraries. We compared the diagnostic scores and diagnostic confidence of the complete MRI protocol composed of the conventional sequences and the ZTE DL images, thereafter named “ZTE DL augmented MRI protocol”, to those of the standard MRI protocol, using conventional sequences alone, and, when available, with those of the X-ray images. Std. MRI vs. ZTE-DL-aug. MRI comparisons were conducted using a mixed-effects model (reader as a random effect) fit on the individual, non-averaged reader-level observations of both readers. Radiographs were assessed by a single reader and were evaluated with a paired Wilcoxon signed-rank test on that reader’s own case-level values. A *p*-value of <0.05 was considered statistically significant. Interreader agreement for both degree of pathologic bone changes and diagnostic confidence was assessed using Cohen’s kappa (k) tests. K values were interpreted as poor (κ < 0), slight (κ = 0.00–0.20), fair (κ = 0.21–0.40), moderate (κ = 0.41–0.60), substantial (κ = 0.61–0.80), or near-perfect (κ = 0.81–1.00) [27].

## 3. Results

The cohort encompassed a total of 59 datasets of both hands and feet (feet = 22, hands = 37) of 40 patients with an average age of 51.79 years (SD ± 11.84 years, 58% women (*n* = 34), 42% men (*n* = 25)). (Table 3). In 22 cases, additional X-rays were available (hands: *n* = 12, feet: *n* = 10; see Methods for the MR-radiograph time interval).

**Figure 1:**
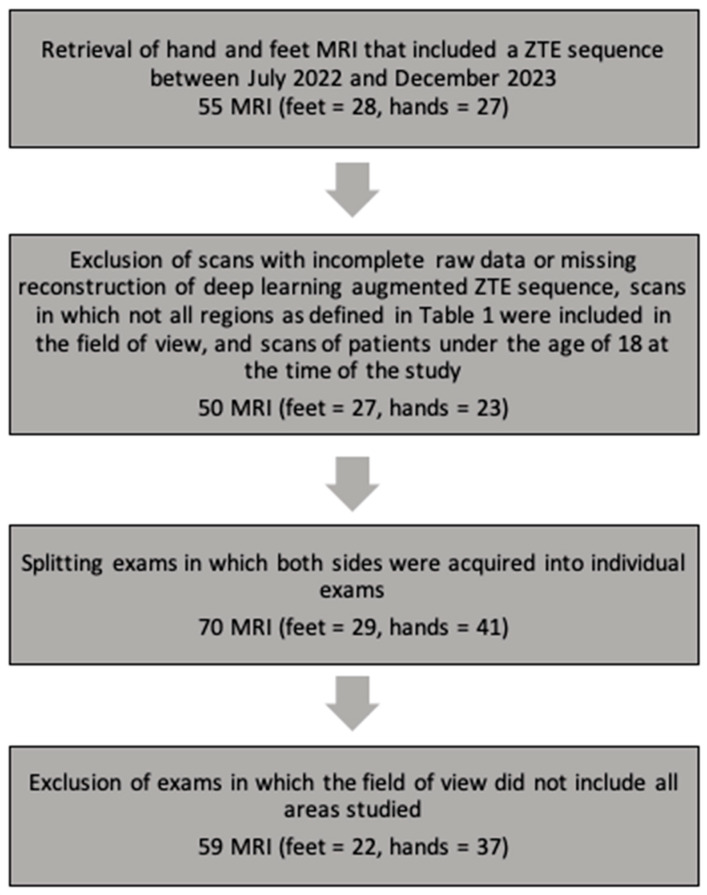
Flow chart from initial retrieval to the final study cohort of 59 datasets (feet = 22, hands = 37), between July 2022 and December 2023. See flow chart for case counts and exclusion criteria at each step.

The study cohort assembly from initial retrieval to the final study cohort can be seen in Figure 1.

### 3.1. Degree of Pathologic Bone Changes

**Figure 2:**
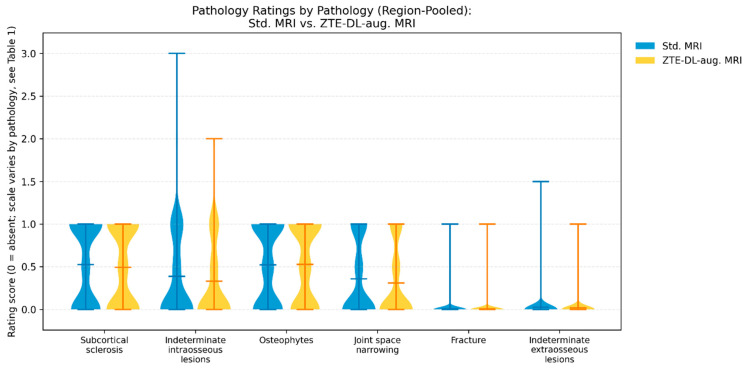
Violin plot of pathology-rating scores by pathology (region-pooled), Std. MRI vs. ZTE-DL-aug. MRI (*n* = 59). Corresponds to Table 4a.

**Figure 3:**
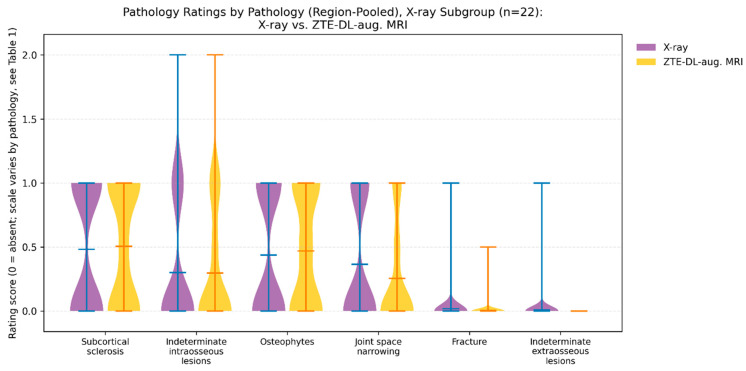
Violin plot of pathology-rating scores by pathology (region-pooled), X-ray vs. ZTE-DL-aug. MRI, restricted to the 22-case X-ray subgroup used in the Bland–Altman analysis (Figure 4).

**Figure 4:**
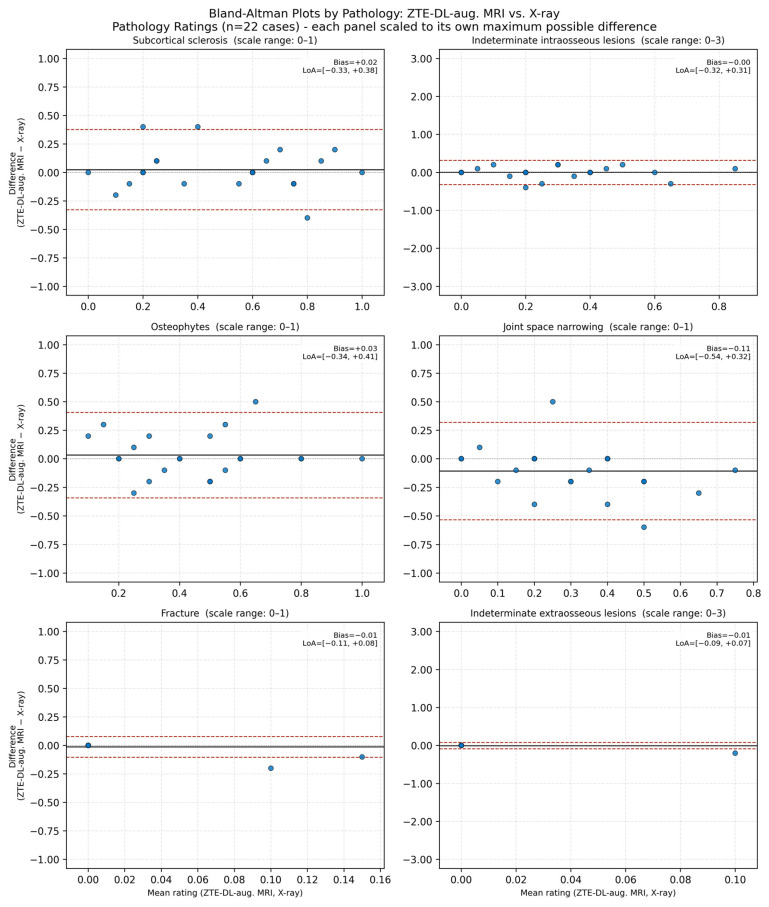
Bland–Altman plots by pathology comparing ZTE-DL-aug. MRI and X-ray pathology-rating scores in the 22-case X-ray subgroup, one panel per pathology (mean rating across the 5 anatomical regions per case). Solid line: mean bias; dashed lines: 95% limits of agreement. The difference axis in each panel is scaled to the maximum possible difference for that pathology’s own rating scale, which differs by pathology: ±1 for subcortical sclerosis, osteophytes, joint space narrowing, and fracture (assessed as present/absent), and ±3 for indeterminate intraosseous and extraosseous lesions (assessed on a 4-point Likert scale).

Mixed-effects model analysis revealed no significant differences in assessment of pathologic bone change (*p*-values > 0.05) when comparing the standard and the ZTE DL augmented MRI protocol. In the subgroup analysis of patients with available radiographs, small absolute differences in Likert scales were found that were statistically significant for osteophytes, subcortical sclerosis, fracture, joint space narrowing, and indeterminate extraosseous lesions. For example, osteophytes were rated on radiographs as 0.44 and on the ZTE DL augmented MRI protocol as 0.56, suggesting ZTE DL augmented MRI may improve the reader’s subjective diagnostic confidence for subtle osseous changes compared to radiographs, though this cannot be interpreted as higher diagnostic sensitivity in the absence of a reference standard. Interreader agreement was moderate to near-perfect for assessment of osseous changes in the standard MRI protocol with kappa between 0.57 and 0.83, and fair to near-perfect for the standard MRI protocol with the addition of the ZTE DL sequence with kappa between 0.33 and 1.00. Agreement for fracture assessment with the ZTE DL sequence was fair (kappa = 0.33, 95% CI crossing zero); fractures were a rare finding in this cohort, which limits the stability of this kappa estimate. Details can be seen in Table 4a,b and Figure 2. The pathology-rating distribution in the X-ray subgroup is shown in Figure 3, with per-pathology Bland–Altman comparisons to X-ray shown in Figure 4.

### 3.2. Diagnostic Confidence

**Figure 5:**
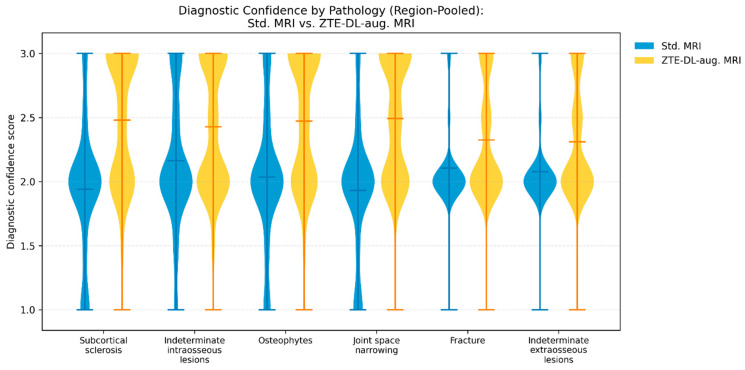
Violin plot of diagnostic-confidence scores by pathology (region-pooled), Std. MRI vs. ZTE-DL-aug. MRI (*n* = 59). Corresponds to Table 5a.

**Figure 6:**
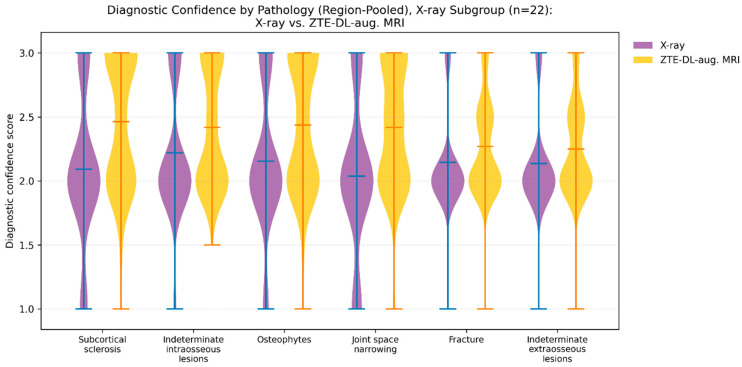
Violin plot of diagnostic-confidence scores by pathology (region-pooled), X-ray vs. ZTE-DL-aug. MRI, restricted to the 22-case X-ray subgroup.

**Figure 7:**
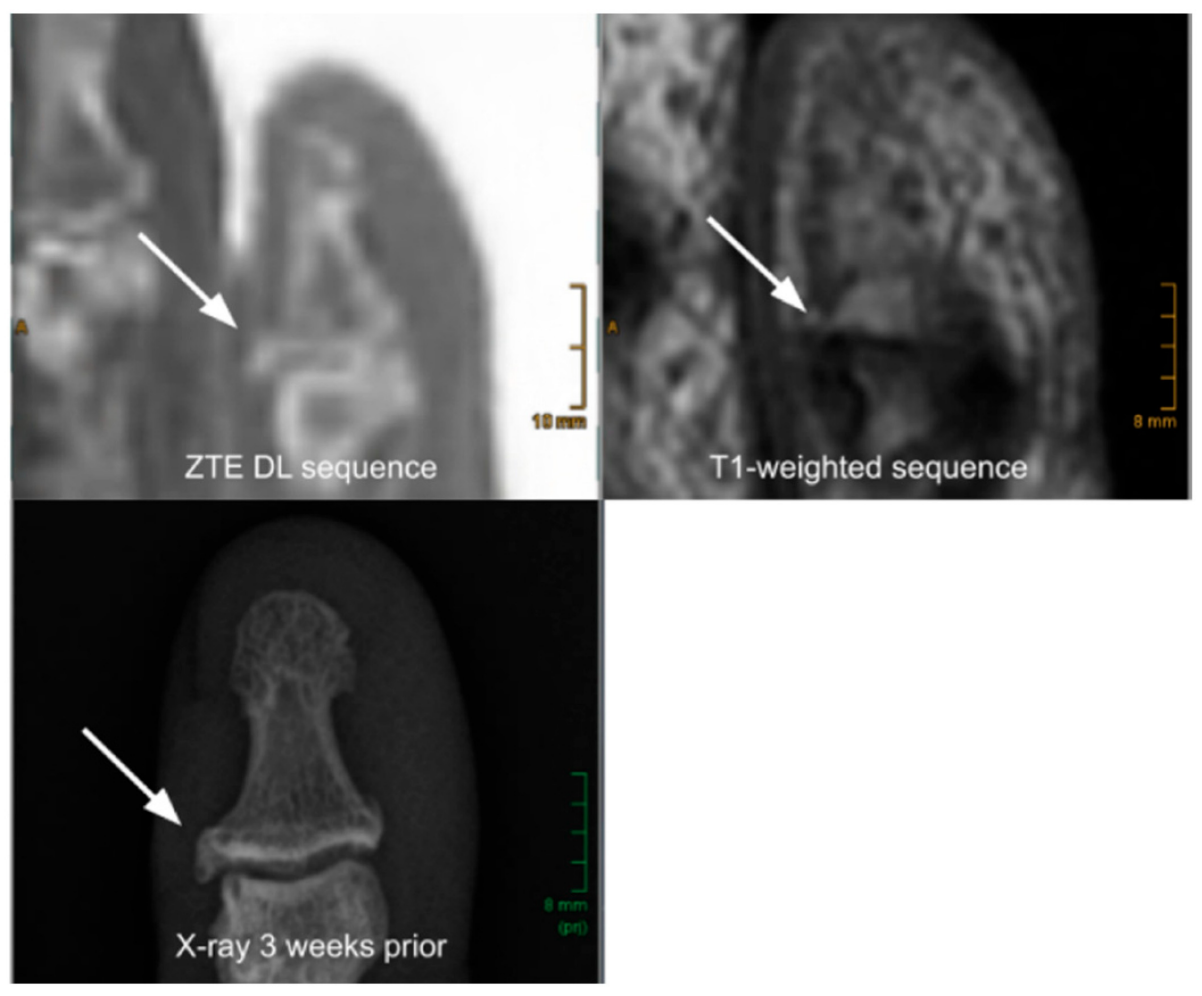
Sixty-year-old male with osteoarthritis of the hand and pain in the proximal and distal interphalangeal joints, imaged with coronal ZTE DL (**left**) and T1-weighted MRI (**right**), with a corresponding radiograph obtained 3 weeks earlier (**bottom**). An osteophyte (white arrow) at the distal interphalangeal joint is more clearly depicted on ZTE DL than on T1-weighted imaging.

**Figure 8:**
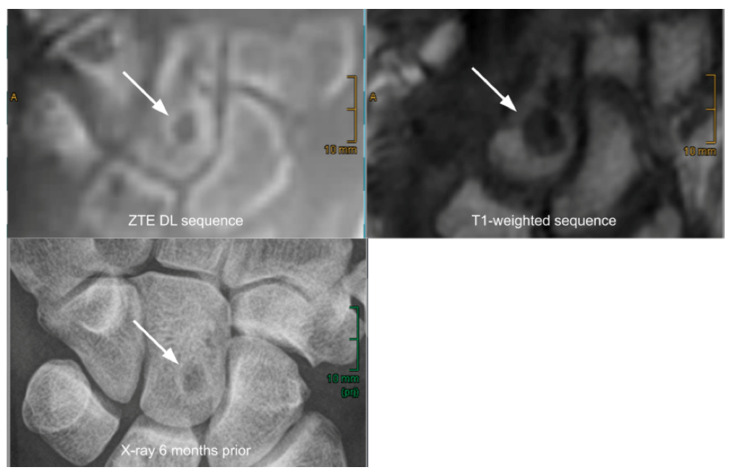
Forty-two-year-old male with swollen hands, in whom MRI was performed to assess the soft tissues, imaged with coronal ZTE DL (**left**) and T1-weighted (**right**) MRI, with a corresponding radiograph obtained 6 months earlier (**bottom**). An intraosseous lesion in the Os capitatum, most probably in keeping with fibro-osseous rests, is more clearly visualized on ZTE DL, with an overall CT-like appearance, than on T1-weighted imaging.

**Figure 9:**
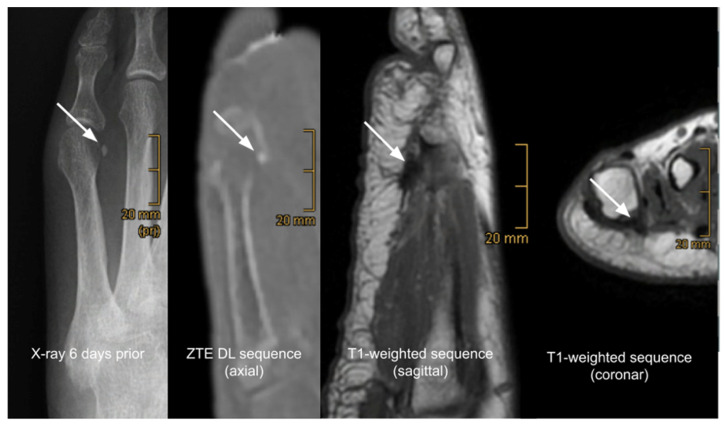
Seventy-three-year-old female with suspected Morton neuroma, in whom X-ray and MRI were performed, imaged with ZTE DL and coordination-matched sagittal and coronal T1-weighted MRI (no axial T1-weighted images were acquired), with a corresponding radiograph obtained 6 days earlier. An ossicle at the metatarsophalangeal joint V is visible on the radiograph and on ZTE DL, but not on the T1-weighted sequences.

**Figure 10:**
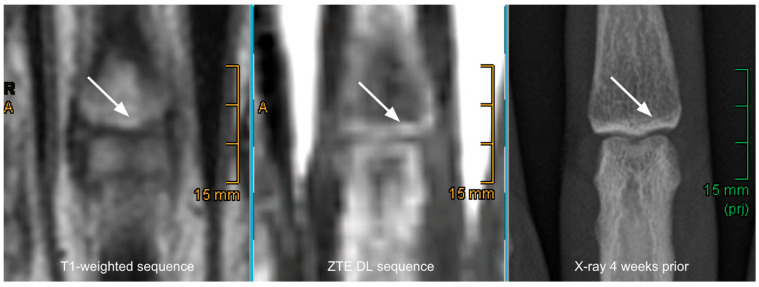
Sixty-year-old male with finger joint osteoarthritis, imaged with ZTE DL and T1-weighted MRI, with a corresponding radiograph obtained 4 weeks earlier. Potential subchondral sclerosis is better visualized at the third proximal interphalangeal joint with ZTE DL compared to T1-weighted MRI.

**Figure 11:**
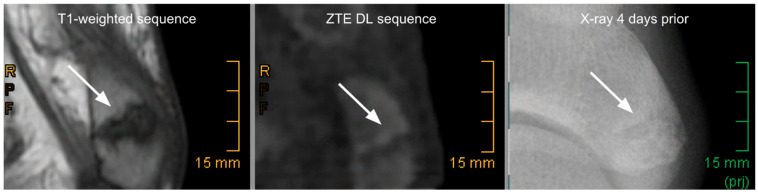
Eighteen-year-old male whose radiograph showed a suspected traumatic injury of the os naviculare, with os tibiale externum as a differential diagnosis, imaged with ZTE DL and T1-weighted MRI, with a corresponding radiograph obtained 4 days earlier. MRI with ZTE DL confirmed an os tibiale externum rather than a fracture.

**Figure 12:**
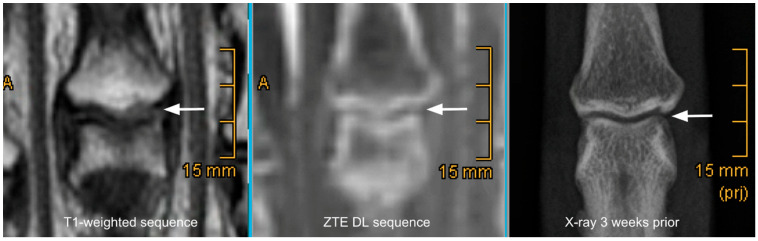
Forty-nine-year-old female with recurring arthralgias and edema in the hands, imaged with ZTE DL and T1-weighted MRI, with a corresponding radiograph obtained 3 weeks earlier. The joint space at the third proximal interphalangeal joint is better visualized using ZTE DL.

The integration of ZTE DL demonstrated higher diagnostic confidence across all pathologic bone changes evaluated compared to the standard MRI protocol (*p*-values < 0.05), and, overall, compared to X-ray (*p* < 0.001); this X-ray comparison did not reach significance for indeterminate extraosseous lesions specifically (*p* = 0.061). Interreader agreement was substantial for assessment of osseous changes on the standard protocol with kappa between 0.62 and 0.71, and moderate to substantial on the ZTE DL augmented MRI protocol with kappa between 0.59 and 0.68. Details can be seen in Table 5a,b and Figure 5. The diagnostic-confidence distribution in the X-ray subgroup is shown in Figure 6. An example of improved visibility of an osteophyte is shown in Figure 7 and of an intraosseous lesion in Figure 8. Additionally, an example of an ossicle at the metatarsophalangeal joint V, which could not be visualized in the accompanying T1-weighted sequences, is shown in Figure 9, of subchondral sclerosis in Figure 10, of an os tibiale externum in Figure 11, and of improved joint space visualization in Figure 12.

### 3.3. Subgroup Analysis by Anatomic Region

**Figure 13:**
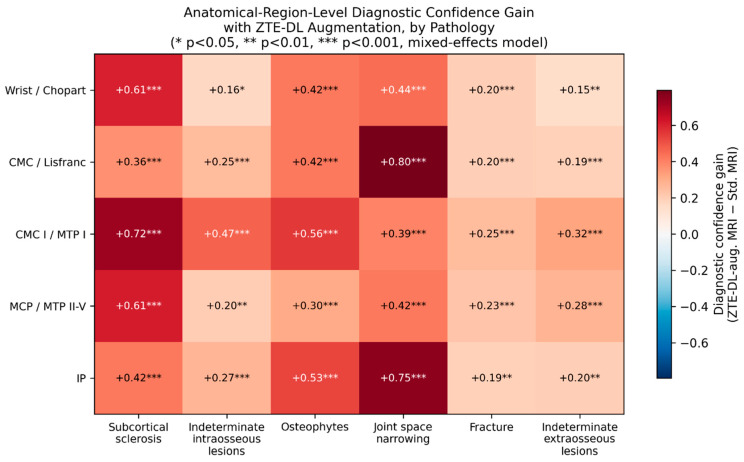
Anatomical-region-level diagnostic-confidence gain with ZTE-DL augmentation (ZTE-DL-aug. MRI mean minus Std. MRI mean), by pathology. Rows: five anatomical regions (Wrist/Chopart, CMC/Lisfranc, CMC I/MTP I, MCP/MTP II-V, IP). Columns: six pathologies. Cell values are the confidence gain; asterisks denote significance from a mixed-effects model (reader as a random effect)—* *p* < 0.05, ** *p* < 0.01, *** *p* < 0.001. Full numeric detail, including Holm–Bonferroni-corrected *p*-values and inter-reader kappa, is provided in supplementary Table S5.

Anatomical-region-level subgroup analysis revealed no statistically significant differences in pathology ratings after Holm–Bonferroni correction, as detailed in supplementary Table S4. In contrast, diagnostic-confidence gain with ZTE DL augmentation was positive and statistically significant across all five anatomical regions and six pathologic bone changes when compared to the standard MRI protocol, and mostly higher when compared to radiographs (12/30 region-by-pathology comparisons, *p* < 0.05), as visualized in a heatmap in the main text (Figure 13); full numeric detail, including inter-reader kappa, is available in the supplemental material (supplementary Table S5.).

## 4. Discussion

Our study demonstrates that the integration of DL-reconstructed ZTE sequences into standard MRI protocols for the hands and feet significantly increases diagnostic confidence in the assessment of bone changes compared with conventional MRI sequences alone. To the best of our knowledge, this is the first study to evaluate the addition of ZTE DL sequences to the conventional MRI protocol for assessment of small joints in hands and feet using a 1.5 T MRI scanner. It is important to note that diagnostic confidence is not equivalent to diagnostic accuracy; ZTE DL reconstruction may increase reader confidence while potentially introducing reconstruction-related bias, such as noise suppression, edge sharpening, or artificial-looking structures, and that warrants further investigation.

These results are concordant with previous studies on the use of ZTE DL for the evaluation of larger joints, such as the shoulder [7,18] and the knee [8,9]. Our study extends the body of evidence that ZTE DL sequences may help in the assessment of osseous structures even in small joints at 1.5 T. 1.5 T MRI scanners are widely available, and therefore DL-optimized ZTE sequences can meet a widespread need for optimized bone depiction at lower field strength. In this context, particularly our finding of higher diagnostic confidence for indeterminate intraosseous lesions (erosions and intraosseous ganglia) with the addition of ZTE DL sequences to a conventional MSK MRI protocol of hands and feet at 1.5 T is of interest, as MRI examinations are the preferred imaging modality for the assessment of these lesions [28].

Our ZTE DL sequence had an average acquisition time of 2 min for the hand protocol of both hands and 2 min and 20 s for the foot protocol of one foot. In comparison, the total scan time of the protocols used was about 15–20 min. An additional CT and/or radiograph would easily take more than 2 min for patient repositioning and acquisition. ZTE DL sequences can thus be considered a viable addition to conventional MRI protocols of hands and feet at 1.5 T, especially if the evaluation of pathologic bone changes is of particular interest and prior radiographs or CT are not available.

An important advantage of the ZTE DL sequence is its 3D acquisition mode, which helps in multiplanar evaluation of small osseous changes in the hands and feet. As late-stage rheumatoid disease changes such as subluxations, swan-neck deformities or boutonniere deformities become rarer with the widespread onset of biologic disease-modifying anti-rheumatic drug treatment [29], assessment of these small early osseous changes becomes even more relevant. Future advancements in deep learning could also allow fused imaging of the most peripheral nerves and visualization of their relation to the adjacent small bones and joints, as it is already done in more proximal anatomies, for example in the case of humeral fractures and their relation to the radial nerve [30]. ZTE DL sequences could also improve the possibility of measuring bone loss in the small bones of hands and feet due to rheumatological diseases, a method already used in bigger joints such as the shoulder [31]. Furthermore, ZTE DL may improve the detection of subclinical injuries in smaller joints and bones that may later predispose to osteoarthritis [32]. Finally, in a few selected regions, individual parameters differed between ZTE and ZTE DL, notably that joint space narrowing was reported less in two joint groups. This finding suggests that subtle deep-learning-related reconstruction biases, such as artificial suppression or amplification of imaging features, and/or slight AI-generated modifications of image contrast [33], could be a concern when using ZTE DL in small joints and hands.

There are limitations to this study. This study lacks a reference standard for pathologic bone changes, as this would be a prerequisite for a classic diagnostic performance study. The reconstruction algorithm used in this study (AIR™ Recon DL) is proprietary, which limits full architectural transparency; additionally, future work using open, well-documented reconstruction architectures such as MobileNet, multilayer perceptrons, or ResNet, which have demonstrated efficacy in MRI reconstruction, would allow fuller disclosure of model design, training data, and hyperparameters. As one example of such a transparent, well-documented architecture from a related imaging-diagnosis context, a flexible multi-channel deep network combining texture and spatial features was recently applied to diagnosing COVID-19 variants in lung CT scans [34]. In the absence of a reference standard, we cannot exclude the possibility that ZTE DL reconstruction introduces subtle “hallucination” or over-enhancement artifacts that could increase reader confidence without a corresponding increase in diagnostic accuracy; this risk should be considered when interpreting our results.

The variable inter-reader agreement for ZTE DL warrants further evaluation before clinical adoption. To account for that, we performed a subgroup analysis comparing the ZTE DL augmented MRI protocol to available radiographs. This radiograph comparison should be regarded as exploratory, given that it was available only in a subgroup of cases and with a variable time interval to MRI. This analysis, including 22 scans, showed that diagnostic confidence in assessing pathologic bone changes in the hands and feet continued to be higher in the ZTE DL augmented MRI protocol when compared to radiographs.

Another potential limitation for clinical use is the availability of ZTE DL sequences and reconstructions in a clinical setting. This study should be regarded as an exploratory pilot/feasibility study given its sample size, particularly for subgroup analyses of rarer pathologies such as fractures and indeterminate extraosseous lesions (ossicles or soft tissue calcifications); larger prospective studies are required to confirm these findings. However, this study shows that if these sequences are available, they can be used for the assessment of the bone in small joints without the need for additional ionizing radiation. This can be useful both in first-line assessment of small joints in MRI and in patients who have already had many prior examinations with ionizing radiation or might need follow-up, in which case ZTE DL sequences would be appropriate.

## 5. Conclusions

In conclusion, the integration of deep learning–reconstructed zero-echo-time (ZTE) sequences into 1.5-T MRI protocols for imaging of the hands and feet resulted in significantly higher diagnostic confidence for evaluating a range of bone pathologies compared with conventional MRI sequences alone. The addition of ZTE DL increased reader diagnostic confidence for osseous assessment of the hands and feet, but further validation against a reference standard is required to determine diagnostic accuracy. Our findings suggest improved confidence, but prospective studies with a reference standard are needed to confirm whether ZTE DL can safely reduce radiation exposure.

## Figures and Tables

**Table 1:** Joint region definitions and maximum joints per region.

Evaluated Joint Regions for Hands	Evaluated Joint Regions for Feet
Radioulnocarpal (radiocarpal and distal radioulnar joint) and proximal Gilula arc	Chopart
CMC II-V and distal Gilula arcs	Lisfranc
CMC I	MTP I
MCP I-V	MTP II-V
IP I-V	IP I-V

**Table 2:** Scan parameters of the zero echo time (ZTE) sequence.

Sequence Type	ZTE
MRI acquisition type	3D
Echo time (ms)	0.016
Repetition time (ms)	Hands: 482 Feet: 445
Slice thickness (mm)	Hands: 2 Feet: 1.2
Spacing between slices	1
Acquisition matrix	Hands: 224 × 224 Feet: 200 × 200
Flip angle (°)	1
Pixel bandwidth (kHz)	488
Number of averages	4
Field of view (mm)	100
Coils	Hands: 19-channel head and neck coil (*n* = 32), 16-channel FM (*n* = 4) or FL coil (*n* = 1) Feet: 8-channel foot and ankle coil

**Table 3:** Demographics of study population. As each scan was evaluated separately in case of bilateral scans, the numbers refer to total scans and not total patients.

Total Number of MRI Scans Evaluated	59 (Feet = 22, Hands = 37)
Female	34 (58%)
Male	25 (42%)
Average age (years)	51.79 (standard deviation: 11.84)

**Table 4:** (**a**) Evaluation of integrating ZTE DL MRI sequences into a standard MRI protocol in evaluating pathologic bone changes: Std. MRI vs. ZTE-DL-aug. MRI. Average rating for each pathologic bone change with average standard deviation (SD) and average kappa interreader agreement (kappa), for the evaluation using only the standard MRI protocol (Std MRI) and with the aid of the ZTE DL sequence (ZTE-DL-aug. MRI). This comparison uses a mixed-effects model (reader as a random effect) fit on the individual, non-averaged reader-level ratings, reported as the z-statistic and *p*-value, additionally reported after Holm–Bonferroni correction across the 6 pathologies. Kappa 95% confidence intervals are clipped to the valid [−1, 1] range where the underlying normal-approximation calculation would otherwise extend beyond it. (**b**) Evaluation of integrating ZTE DL MRI sequences into a standard MRI protocol in evaluating pathologic bone changes: X-ray vs. ZTE-DL-aug. MRI. Average rating for each pathologic bone change with average standard deviation (SD) for the evaluation using conventional radiographs (X-ray) and with the aid of the ZTE DL sequence (ZTE-DL-aug. MRI), restricted to the *N* = 22 X-ray subgroup; the ZTE-DL-aug. MRI mean/SD shown here are computed on this same matched subgroup, not the full cohort. This comparison uses a paired Wilcoxon signed-rank test, reported as the W statistic and *p*-value, additionally reported after Holm–Bonferroni correction across the 6 pathologies.

**(a)**									
**Feature**	**Mean (Std. MRI)**	**SD (Std. MRI)**	**Kappa (Std. MRI) 95% CI**	**Mean (ZTE-DL-aug. MRI)**	**SD (ZTE-DL-aug. MRI)**	**Kappa (ZTE-DL-aug. MRI) 95% CI**	**Z Statistic**	***p*-Value**	**Holm-Adjusted *p*-Value**
Indeterminate intraosseous lesions	0.39	0.54	0.66 [0.55, 0.78]	0.33	0.50	0.58 [0.44, 0.73]	−1.59	0.112	0.560
Osteophytes	0.52	0.46	0.61 [0.54, 0.67]	0.53	0.46	0.62 [0.56, 0.69]	0.17	0.864	1.000
Subcortical sclerosis	0.53	0.47	0.70 [0.58, 0.82]	0.49	0.46	0.66 [0.59, 0.73]	−1.14	0.254	1.000
Fracture	0.01	0.09	0.83 [0.59, 1.00]	0.01	0.08	0.33 [−0.21, 0.87]	0.30	0.762	1.000
Joint space narrowing	0.36	0.44	0.60 [0.52, 0.67]	0.31	0.42	0.55 [0.45, 0.66]	−1.94	0.052	0.313
Indeterminate extraosseous lesions	0.03	0.17	0.57 [0.20, 0.94]	0.02	0.13	1.00 [1.00, 1.00]	−1.01	0.314	1.000
**(b)**									
**Feature**	**Mean (ZTE-DL-aug. MRI)**	**SD (ZTE-DL-aug. MRI)**	**Mean (X-Ray)**		**SD (X-Ray)**	**W Statistic**	***p*** **-Value**		**Holm-Adjusted** ***p*** **-Value**
Indeterminate intraosseous lesions	0.24	0.24	0.30		0.51	131.50	0.167		0.167
Osteophytes	0.56	0.29	0.44		0.50	28.50	<0.001		<0.001
Subcortical sclerosis	0.47	0.28	0.48		0.51	12.00	<0.001		<0.001
Fracture	0.01	0.06	0.02		0.13	8.50	<0.001		<0.001
Joint space narrowing	0.28	0.23	0.36		0.49	41.50	<0.001		<0.001
Indeterminate extraosseous lesions	0.02	0.06	0.01		0.10	25.00	<0.001		<0.001

**Table 5:** (**a**) Descriptive and probabilistic statistics for each diagnostic confidence evaluation: Std. MRI vs. ZTE-DL-aug. MRI. Average rating (mean) for overall diagnostic confidence and diagnostic confidence of each pathologic bone change reading with average standard deviation (SD) and average kappa interreader agreement (kappa), for the evaluation using only the standard protocol (Std. MRI) and with the aid of the ZTE DL sequence (ZTE-DL-aug. MRI). This comparison uses a mixed-effects model (reader as a random effect) fit on the individual, non-averaged reader-level scores, reported as the z-statistic and *p*-value, additionally reported after Holm–Bonferroni correction across the 7 rows (overall + 6 pathologies). Kappa 95% confidence intervals are clipped to the valid [−1, 1] range where the underlying normal-approximation calculation would otherwise extend beyond it. (**b**) Descriptive and probabilistic statistics for each diagnostic confidence evaluation: X-ray vs. ZTE-DL-aug. MRI. Average rating (mean) for overall diagnostic confidence and diagnostic confidence of each pathologic bone change reading with average standard deviation (SD) for the evaluation using conventional radiographs (X-ray) and with the aid of the ZTE DL sequence (ZTE-DL-aug. MRI), restricted to the *N* = 22 X-ray subgroup; the ZTE-DL-aug. MRI mean/SD shown here are computed on this same matched subgroup, not the full cohort. This comparison uses a paired Wilcoxon signed-rank test, reported as the W statistic and *p*-value, additionally reported after Holm–Bonferroni correction across the 7 rows (overall + 6 pathologies).

**(a)**									
**Feature**	**Mean (Std. MRI)**	**SD (Std. MRI)**	**Kappa (Std. MRI) 95% CI**	**Mean (ZTE-DL-aug. MRI)**	**SD (ZTE-DL-aug. MRI)**	**Kappa (ZTE-DL-aug. MRI) 95% CI**	**Z Statistic**	***p*-Value**	**Holm-Adjusted *p*-Value**
Diagnostic confidence overall	2.04	0.49	0.66 [0.62, 0.71]	2.42	0.50	0.63 [0.60, 0.66]	29.88	<0.001	<0.001
Diagnostic confidence Indeterminate intraosseous lesions	2.16	0.54	0.64 [0.58, 0.71]	2.43	0.50	0.63 [0.57, 0.69]	8.32	<0.001	<0.001
Diagnostic confidence Osteophytes	2.04	0.55	0.64 [0.62, 0.66]	2.47	0.54	0.67 [0.62, 0.72]	13.19	<0.001	<0.001
Diagnostic confidence Subcortical sclerosis	1.94	0.55	0.66 [0.59, 0.73]	2.48	0.54	0.68 [0.57, 0.79]	16.30	<0.001	<0.001
Diagnostic confidence Fracture	2.11	0.33	0.71 [0.50, 0.91]	2.33	0.44	0.61 [0.56, 0.65]	8.60	<0.001	<0.001
Diagnostic confidence Joint space narrowing	1.93	0.54	0.62 [0.54, 0.70]	2.49	0.50	0.59 [0.52, 0.66]	17.22	<0.001	<0.001
Diagnostic confidence Indeterminate extraosseous lesions	2.08	0.35	0.71 [0.59, 0.82]	2.31	0.44	0.61 [0.58, 0.64]	9.17	<0.001	<0.001
**(b)**									
**Feature**	**Mean (ZTE-DL-aug. MRI)**	**SD (ZTE-DL-aug. MRI)**		**Mean (X-Ray)**	**SD (X-Ray)**	**W Statistic**	***p *** **-Value**		**Holm-Adjusted** ***p*** **-Value**
Diagnostic confidence overall	2.54	0.32		2.13	0.55	22.50	<0.001		<0.001
Diagnostic confidence Indeterminate intraosseous lesions	2.53	0.38		2.22	0.54	133.50	0.040		0.081
Diagnostic confidence Osteophytes	2.59	0.38		2.15	0.60	13.00	<0.001		<0.001
Diagnostic confidence Subcortical sclerosis	2.62	0.25		2.09	0.62	37.00	<0.001		0.001
Diagnostic confidence Fracture	2.41	0.40		2.15	0.39	77.00	0.007		0.021
Diagnostic confidence Joint space narrowing	2.55	0.35		2.04	0.64	31.50	<0.001		<0.001
Diagnostic confidence Indeterminate extraosseous lesions	2.39	0.37		2.14	0.43	111.00	0.061		0.081

## Data Availability

Anonymized, case-level reader scores, summary datasets, and the analysis code are available from the corresponding author upon request. The underlying identifiable imaging data are not publicly available due to patient privacy restrictions.

## Supplementary Materials

The following supporting information can be downloaded at: https://www.mdpi.com/article/10.3390/diagnostics16152363/s1, Table S1. Scan parameters of the foot protocol (other than the ZTE sequence acquisition) using an 8-channel foot and ankle coil. Table S2. Scan parameters of the hand protocol (other than the ZTE sequence acquisition) using a 19-channel head and neck coil, 16-channel FM or FL coil. Table S3. Sensitivity analysis of the diagnostic-confidence improvement with ZTE DL augmentation, by coil used (all cases, the dedicated-coils-only subset, and each individual coil). Wilcoxon W statistic and *p*-value are from a paired signed-rank test on per-case mean diagnostic confidence (Std. MRI vs. ZTE-DL-aug. MRI) within each group; a Holm-Bonferroni-corrected *p*-value is reported alongside (correction applied across the 5 groups with a valid test). ‘n/a’ indicates the group had too few cases (the single-case 16-channel FL coil group) for a meaningful test. Table S4. Anatomical-subgroup detail of pathology-rating scores. For each of the five anatomical regions and six pathologies, mean pathology-rating scores are reported for the standard MRI protocol (Std.) and the ZTE DL-augmented MRI protocol (ZTE-DL), together with the resulting gain (ZTE-DL minus Std.), a mixed-effects model test statistic and p-value (reader as a random effect, fit on individual reader-level values), a Holm-Bonferroni-corrected *p*-value (correction applied across all 30 region-by-pathology comparisons within the rating family), and the inter-reader Cohen’s kappa (95% CI) for the rating score. Kappa 95% confidence intervals are clipped to the valid [−1, 1] range. ‘n/a’ indicates a kappa could not be computed due to zero variance in that rating within the given region. Table S5. Anatomical-subgroup detail of diagnostic-confidence scores. For each of the five anatomical regions and six pathologies, mean diagnostic-confidence scores are reported for the standard MRI protocol (Std.) and the ZTE DL-augmented MRI protocol (ZTE-DL), together with the resulting gain (ZTE-DL minus Std.), a mixed-effects model test statistic and *p*-value (reader as a random effect, fit on individual reader-level values), a Holm-Bonferroni-corrected *p*-value (correction applied across all 30 region-by-pathology comparisons within the diagnostic-confidence family), and the inter-reader Cohen’s kappa (95% CI) for the diagnostic-confidence score. Kappa 95% confidence intervals are clipped to the valid [−1, 1] range.

## References

[1] Stensby J.D., Fox M.G., Nacey N., Blankenbaker D.G., Frick M.A., Jawetz S.T., Raizman N.M., Said N., Stephens L.A., Expert Panel on Musculoskeletal Imaging (2024). ACR appropriateness criteria^®^ chronic hand and wrist pain: 2023 update. J. Am. Coll. Radiol..

[2] Tafur M., Bencardino J.T., Roberts C.C., Appel M., Bell A.M., Gyftopoulos S., Metter D.F., Mintz D.N., Morrison W.B., Expert Panel on Musculoskeletal Imaging (2020). ACR Appropriateness Criteria^®^ Chronic Foot Pain. J. Am. Coll. Radiol..

[3] Colebatch A.N., Edwards C.J., Østergaard M., van der Heijde D., Balint P.V., D’Agostino M.-A., Forslind K., Grassi W., Haavardsholm E.A., Haugeberg G. (2013). EULAR recommendations for the use of imaging of the joints in the clinical management of rheumatoid arthritis. Ann. Rheum. Dis..

[4] Ahlawat S., Ghasemi A., Fayad L.M. (2025). How I do it: MRI of the bone with marrow-specific sequences. Radiology.

[5] Teixeira P.A.G., Kessler H., Morbée L., Douis N., Boubaker F., Gillet R., Blum A. (2025). Mineralized tissue visualization with MRI: Practical insights and recommendations for optimized clinical applications. Diagn. Interv. Imaging.

[6] Deininger-Czermak E., Euler A., Franckenberg S., Finkenstaedt T., Villefort C., Gascho D., Guggenberger R. (2022). Evaluation of ultrashort echo-time (UTE) and fast-field-echo (FRACTURE) sequences for skull bone visualization and fracture detection—A postmortem study. J. Neuroradiol..

[7] Ensle F., Kaniewska M., Lohezic M., Guggenberger R. (2024). Enhanced bone assessment of the shoulder using zero-echo time MRI with deep-learning image reconstruction. Skelet. Radiol..

[8] Ensle F., Abel F., Lohezic M., Obermüller C., Guggenberger R. (2024). Deep learning reconstruction for optimized bone assessment in zero echo time MR imaging of the knee. Eur. J. Radiol..

[9] Kaniewska M., Deininger-Czermak E., Lohezic M., Ensle F., Guggenberger R. (2023). Deep learning convolutional neural network reconstruction and radial k-space acquisition MR technique for enhanced detection of retropatellar cartilage lesions of the knee joint. Diagnostics.

[10] Kaniewska M., Kuhn D., Deininger-Czermak E., Wolharn L., Finkenstaedt T., Guggenberger R. (2023). 3D zero-echo time and 3D T1-weighted gradient-echo MRI sequences as an alternative to CT for the evaluation of the lumbar facet joints and lumbosacral transitional vertebrae. Acta Radiol..

[11] Deininger-Czermak E., Villefort C., von Knebel Doeberitz N., Franckenberg S., Kälin P., Kenkel D., Gascho D., Piccirelli M., Finkenstaedt T., Thali M.J. (2021). Comparison of MR ultrashort echo time and optimized 3D-multiecho in-phase sequence to computed tomography for assessment of the osseous craniocervical junction. J. Magn. Reson. Imaging.

[12] Deininger-Czermak E., Gascho D., Franckenberg S., Kälin P., Blüthgen C., Villefort C., Thali M.J., Guggenberger R. (2023). Added value of ultra-short echo time and fast field echo using restricted echo-spacing MR imaging in the assessment of the osseous cervical spine. Radiol. Med..

[13] Kaniewska M., Zecca F., Obermüller C., Ensle F., Deininger-Czermak E., Lohezic M., Guggenberger R. (2025). Deep learning reconstruction of zero-echo time sequences to improve visualization of osseous structures and associated pathologies in MRI of cervical spine. Insights Imaging.

[14] Huber F.A., Schumann P., von Spiczak J., Wurnig M.C., Klarhöfer M., Finkenstaedt T., Bedogni A., Guggenberger R. (2020). Medication-related osteonecrosis of the jaw-comparison of bone imaging using ultrashort echo-time magnetic resonance imaging and cone-beam computed tomography. Investig. Radiol..

[15] Do S., Song K.D., Chung J.W. (2020). Basics of deep learning: A radiologist’s guide to understanding published radiology articles on deep learning. Korean J. Radiol..

[16] Ensle F., Kaniewska M., Tiessen A., Lohezic M., Getzmann J.M., Guggenberger R. (2023). Diagnostic performance of deep learning-based reconstruction algorithm in 3D MR neurography. Skelet. Radiol..

[17] Rajpurkar P., Irvin J., Ball R.L., Zhu K., Yang B., Mehta H., Duan T., Ding D., Bagul A., Langlotz C.P. (2018). Deep learning for chest radiograph diagnosis: A retrospective comparison of the CheXNeXt algorithm to practicing radiologists. PLoS Med..

[18] Kaniewska M., Deininger-Czermak E., Getzmann J.M., Wang X., Lohezic M., Guggenberger R. (2023). Application of deep learning-based image reconstruction in MR imaging of the shoulder joint to improve image quality and reduce scan time. Eur. Radiol..

[19] Geethanath S., Reddy R., Konar A.S., Imam S., Sundaresan R., D.R. R.B., Venkatesan R. (2013). Compressed sensing MRI: A review. Crit. Rev. Biomed. Eng..

[20] Feng L., Benkert T., Block K.T., Sodickson D.K., Otazo R., Chandarana H. (2017). Compressed sensing for body MRI. J. Magn. Reson. Imaging.

[21] Tsao J., Kozerke S. (2012). MRI temporal acceleration techniques. J. Magn. Reson. Imaging.

[22] Knoll F., Hammernik K., Zhang C., Moeller S., Pock T., Sodickson D.K., Akcakaya M. (2020). Deep-learning methods for parallel magnetic resonance imaging reconstruction: A survey of the current approaches, trends, and issues. IEEE Signal Process Mag..

[23] Quan T.M., Nguyen-Duc T., Jeong W.-K. (2018). Compressed sensing MRI reconstruction using a generative adversarial network with a cyclic loss. IEEE Trans. Med. Imaging.

[24] Schumann P., Morgenroth S., Huber F.A., Rupp N.J., Del Grande F., Guggenberger R. (2022). Correlation of dynamic contrast-enhanced bone perfusion with morphologic ultra-short echo time MR imaging in medication-related osteonecrosis of the jaw. Dentomaxillofac. Radiol..

[25] Leynes A.P., Yang J., Wiesinger F., Kaushik S.S., Shanbhag D.D., Seo Y., Hope T.A., Larson P.E.Z. (2018). Zero-echo-time and Dixon deep pseudo-CT (ZeDD CT): Direct generation of pseudo-CT images for pelvic PET/MRI attenuation correction using deep convolutional neural networks with multiparametric MRI. J. Nucl. Med..

[26] Peters R.D., Harris H., Lawson S. (2020). The Clinical Benefits of AIRTM Recon DL for MR Image Reconstruction.

[27] Landis J.R., Koch G.G. (1977). The measurement of observer agreement for categorical data. Biometrics.

[28] Sudoł-Szopińska I., Teh J., Cotten A. (2021). Rheumatoid hand and other hand-deforming rheumatic conditions. Semin. Musculoskelet. Radiol..

[29] Pfanner S., Poggetti A. (2025). Swan-neck and boutonniere deformity in rheumatoid hand. Hand Clin..

[30] Lin Y., Tan E.T., Campbell G., Breighner R.E., Fung M., Wolfe S.W., Carrino J.A., Sneag D.B. (2025). How I do It: Three-Dimensional MR Neurography and Zero Echo Time MRI for Rendering of Peripheral Nerve and Bone. Radiology.

[31] Thacher R.R., Retzky J.S., Dekhne M.S., Oquendo Y.A., Greditzer H.G. (2023). Current concepts in the measurement of glenohumeral bone loss. Curr. Rev. Musculoskelet. Med..

[32] Cheng K.Y., Moazamian D., Ma Y., Jang H., Jerban S., Du J., Chung C.B. (2023). Clinical application of ultrashort echo time (UTE) and zero echo time (ZTE) magnetic resonance (MR) imaging in the evaluation of osteoarthritis. Skelet. Radiol..

[33] Haller S., Hedderich D., Federau C., Weisstanner C., Edjlali M., van Cauter S., Zaharchuk G. (2025). The current status of AI-accelerated MRI techniques in clinical use. Radiology.

[34] Fekri-Ershad S., Behrouz Dehkordi K. (2025). A flexible multi-channel deep network leveraging texture and spatial features for diagnosing new COVID-19 variants in lung CT scans. Tomography.

