# Supplementary material for: Deep Learning-Augmented Zero Echo Time MRI Increases Diagnostic Confidence for Osseous Assessment in Hand and Foot MRI Protocols
Source: Diagnostics (Basel). 2026 Jul 27;16(15):2363. doi: 10.3390/diagnostics16152363 (PMC13464802; doi:10.3390/diagnostics16152363)
Supplement: Supplementary file 1 [file diagnostics-16-02363-s001.zip › diagnostics-4429091-supplementary.pdf]

Supplementary

Table S1.supp. Scan parameters of the foot protocol (other than the ZTE sequence acquisition) using an 8-channel foot and ankle coil.

Sequence type	T2 Dixon	T2 Dixon	T2 fat-sat	T1
Orientation	sagittal	axial	coronal	sagittal
MRI acquisition type	2D	2D	2D	2D
Echo time (ms)	81.920	89.712	83.160	7.208
Repetition time (ms)	5900	4321	7195	724
Slice thickness (mm)	3.5	3.5	3.5	3.5
Spacing between slices	3.8	4.0	4.0	3.8
Acquisition matrix	272 x 244	192 x 180	212 x 180	260 x 224
Flip angle (°)	160	160	160	160
Pixel bandwidth (kHz)	355.117	244.141	122.07	162.773
Number of averages	2.00	2.00	1.00	1.50
Field of view (mm)	100	100	140	100

Sequence type	T1	Multi-phase postcontrast sequence (TWIST)	T1 Dixon with intravenous contrast	T1 Dixon with intravenous contrast
Orientation	axial	sagittal	axial	sagittal
MRI acquisition type	2D	3D	2D	2D
Echo time (ms)	7.216	2.316	9.696	9.756
Repetition time (ms)	560	4.888	633	722
Slice thickness (mm)	3.5	2.6	3.5	3.5
Spacing between slices	4.0	1.3	4.0	3.8
Acquisition matrix	260 x 212	280 x 240	180 x 180	264 x 232
Flip angle (°)	160	30	160	160
Pixel bandwidth (kHz)	162.773	244.141	244.141	355.117
Number of averages	1.50	0.75	2.00	2.00
Field of view (mm)	100	90	100	100

Table S2.supp. Scan parameters of the hand protocol (other than the ZTE sequence acquisition) using a 19-channel head and neck coil, 16-channel FM or FL coil.

Sequence type	T2 Dixon	PD Dixon	T1	T1
Orientation	coronal	axial	sagittal	axial
MRI acquisition type	2D	2D	2D	2D
Echo time (ms)	87.048	39.104	8.432	7.64
Repetition time (ms)	3569	5129	706	798
Slice thickness (mm)	2.6	4.0	2.6	4.0
Spacing between slices	2.8	4.5	2.8	4.5
Acquisition matrix	188 x 212	188 x 188	260 x 260	260 x 240
Flip angle (°)	160	160	160	160
Pixel bandwidth (kHz)	325.508	122.07	122.07	139.492
Number of averages	3.00	4.00	2.00	2.00
Field of view (mm)	140	100	140	100

Sequence type	Multi-phase postcontrast sequence (TWIST)	T1 Dixon with intravenous contrast	T1 Dixon with intravenous contrast
Orientation	sagittal	axial	coronal
MRI acquisition type	3D	2D	2D
Echo time (ms)	2.368	9.808	9.592
Repetition time (ms)	5.103	637	647
Slice thickness (mm)	2.0	4.0	2.6
Spacing between slices	1.0	4.5	2.8
Acquisition matrix	260 x 260	192 x 188	188 x 212
Flip angle (°)	30	160	160
Pixel bandwidth (kHz)	195.312	122.07	162.754
Number of averages	1.00	4.00	2.00

Field of view (mm)	60	100	140

Table S3.Sensitivity analysis of the diagnostic-confidence improvement with ZTE DL augmentation by coil used (all cases, the dedicated-coils-only subset, and each individual coil). Wilcoxon W statistic and p-value are from a paired signed-rank test on per-case mean diagnostic confidence (Std. MRI vs. ZTE-DL-aug. MRI) within each group; a Holm–Bonferroni-corrected p-value is reported alongside (correction applied across the 5 groups with a valid test). 'n/a' indicates the group had too few cases (the single-case 16-channel FL coil group) for a meaningful test.

Group	N (cases)	Mean DC (Std. MRI)	SD (Std. MRI)	Mean DC (ZTE-DL-aug. MRI)	SD (ZTE-DL-aug. MRI)	Wilcoxon W	Wilcoxon p-value	Holm-adjusted p-value
All cases	59	2.04	0.49	2.42	0.50	14.00	<0.001	<0.001
Dedicated coils only (excludes non-dedicated hand coils)	54	2.06	0.50	2.43	0.50	14.00	<0.001	<0.001
16-channel FL coil	1	1.92	0.35	2.32	0.40	n/a	n/a (n too small)	n/a
16-channel FM coil	4	1.86	0.45	2.22	0.43	0.00	0.125	0.125
19-channel head and neck coil	32	2.04	0.49	2.41	0.51	4.00	<0.001	<0.001
8-channel foot and ankle coil	22	2.08	0.51	2.47	0.50	4.00	<0.001	<0.001

Table S4. Anatomical-subgroup detail of pathology-rating scores. For each of the five anatomical regions and six pathologies, mean pathology-rating scores are reported for the standard MRI protocol (Std.) and the ZTE DL-augmented MRI protocol (ZTE-DL), together with the resulting gain (ZTE-DL minus Std.), a mixed-effects model test statistic and p-value (reader as a random effect, fit on individual reader-level values), a Holm–Bonferroni-corrected p-value (correction applied across all 30 region-by-pathology comparisons within the rating family), and the inter-reader Cohen's kappa (95% CI) for the rating score. Kappa 95% confidence intervals are clipped to the valid [-1, 1] range. 'n/a' indicates a kappa could not be computed due to zero variance in that rating within the given region.

Region	Pathology	Mean rating (Std.)	Mean rating (ZTE-DL)	Rating gain (ZTE-DL - Std.)	Rating test statistic	Rating p-value	Holm-adjusted rating p-value	Kappa rating (Std.) 95% CI	Kappa rating (ZTE-DL) 95% CI
Wrist / Chopart	Subcortical sclerosis	0.75	0.70	-0.05	-0.86	0.388	1.000	0.82 [0.82, 0.82]	0.76 [0.76, 0.76]
Wrist / Chopart	Indeterminate intraosseous lesions	0.40	0.39	-0.01	-0.12	0.908	1.000	0.63 [0.63, 0.63]	0.63 [0.63, 0.63]
Wrist / Chopart	Osteophytes	0.48	0.46	-0.02	-0.26	0.796	1.000	0.55 [0.55, 0.55]	0.62 [0.62, 0.62]
Wrist / Chopart	Joint space narrowing	0.30	0.20	-0.10	-1.82	0.069	1.000	0.61 [0.61, 0.61]	0.41 [-0.82, 1.00]
Wrist / Chopart	Fracture	0.00	0.00	0.00	n/a	n/a	n/a	n/a	n/a

Wrist / Chopart	Indeterminate extraosseous lesions	0.02	0.05	0.03	1.44	0.150	1.000	1.00 [1.00, 1.00]	1.00 [1.00, 1.00]
CMC / Lisfranc	Subcortical sclerosis	0.33	0.38	0.05	0.8	0.422	1.000	0.85 [0.85, 0.85]	0.72 [0.72, 0.72]
CMC / Lisfranc	Indeterminate intraosseous lesions	0.62	0.59	-0.03	-0.37	0.714	1.000	0.78 [0.78, 0.78]	0.74 [0.74, 0.74]
CMC / Lisfranc	Osteophytes	0.65	0.64	-0.01	-0.13	0.894	1.000	0.68 [0.68, 0.68]	0.72 [0.72, 0.72]
CMC / Lisfranc	Joint space narrowing	0.58	0.52	-0.07	-1.02	0.309	1.000	0.70 [0.70, 0.70]	0.77 [0.77, 0.77]
CMC / Lisfranc	Fracture	0.02	0.01	-0.02	-1.0	0.315	1.000	0.66 [-1.00, 1.00]	0.00 [-1.00, 1.00]
CMC / Lisfranc	Indeterminate extraosseous lesions	0.00	0.00	0.00	n/a	n/a	n/a	n/a	n/a
CMC I / MTP I	Subcortical sclerosis	0.70	0.69	-0.02	-0.28	0.781	1.000	0.53 [0.32, 0.74]	0.62 [0.62, 0.62]
CMC I / MTP I	Indeterminate intraosseous lesions	0.30	0.24	-0.06	-0.97	0.331	1.000	0.60 [0.60, 0.60]	0.50 [-0.14, 1.00]
CMC I / MTP I	Osteophytes	0.80	0.76	-0.04	-0.77	0.439	1.000	0.48 [-0.69, 1.00]	0.50 [-0.14, 1.00]
CMC I / MTP I	Joint space narrowing	0.20	0.22	0.02	0.31	0.754	1.000	0.46 [-0.65, 1.00]	0.58 [0.40, 0.75]
CMC I / MTP I	Fracture	0.00	0.02	0.02	1.74	0.082	1.000	n/a	-0.02 [-1.00, 1.00]
CMC I / MTP I	Indeterminate extraosseous lesions	0.03	0.02	-0.02	-0.71	0.478	1.000	0.66 [-1.00, 1.00]	1.00 [1.00, 1.00]
MCP / MTP II-V	Subcortical sclerosis	0.57	0.44	-0.13	-2.06	0.040	1.000	0.73 [0.73, 0.73]	0.67 [0.67, 0.67]
MCP / MTP II-V	Indeterminate intraosseous lesions	0.37	0.25	-0.11	-1.7	0.089	1.000	0.75 [0.75, 0.75]	0.69 [0.69, 0.69]
MCP / MTP II-V	Osteophytes	0.30	0.29	-0.02	-0.28	0.779	1.000	0.66 [0.66, 0.66]	0.64 [0.64, 0.64]
MCP / MTP II-V	Joint space narrowing	0.22	0.12	-0.10	-2.04	0.041	1.000	0.67 [0.67, 0.67]	0.47 [-1.00, 1.00]
MCP / MTP II-V	Fracture	0.00	0.00	0.00	n/a	n/a	n/a	n/a	n/a
MCP / MTP II-V	Indeterminate extraosseous lesions	0.04	0.02	-0.02	-1.15	0.251	1.000	0.79 [0.79, 0.79]	1.00 [1.00, 1.00]

IP	Subcortical sclerosis	0.27	0.25	-0.02	-0.29	0.774	1.000	0.59 [0.59, 0.59]	0.54 [0.26, 0.81]
IP	Indeterminate intraosseous lesions	0.24	0.20	-0.04	-0.75	0.455	1.000	0.48 [-0.19, 1.00]	0.27 [-1.00, 1.00]
IP	Osteophytes	0.38	0.48	0.11	1.67	0.096	1.000	0.69 [0.69, 0.69]	0.64 [0.64, 0.64]
IP	Joint space narrowing	0.49	0.48	-0.02	-0.25	0.803	1.000	0.58 [0.58, 0.58]	0.54 [0.54, 0.54]
IP	Fracture	0.02	0.02	0.00	0.0	1.000	1.000	1.00 [1.00, 1.00]	1.00 [1.00, 1.00]
IP	Indeterminate extraosseous lesions	0.04	0.00	-0.04	-1.39	0.163	1.000	0.00 [-1.00, 1.00]	n/a

Table S5. Anatomical-subgroup detail of diagnostic-confidence scores. For each of the five anatomical regions and six pathologies, mean diagnostic-confidence scores are reported for the standard MRI protocol (Std.) and the ZTE DL-augmented MRI protocol (ZTE-DL), together with the resulting gain (ZTE-DL minus Std.), a mixed-effects model test statistic and p-value (reader as a random effect, fit on individual reader-level values), a Holm–Bonferroni-corrected p-value (correction applied across all 30 region-by-pathology comparisons within the diagnostic-confidence family), and the inter-reader Cohen's kappa (95% CI) for the diagnostic-confidence score. Kappa 95% confidence intervals are clipped to the valid [-1, 1] range.

**Region**	**Pathology**	**Mean DC (Std.)**	**Mean DC (ZTE-DL)**	**Confidence gain (ZTE-DL - Std.)**	**DC test statistic**	**DC p-value**	**Holm-adjusted DC p-value**	**Kappa DC (Std.) 95% CI**	**Kappa DC (ZTE-DL) 95% CI**
Wrist / Chopart	Subcortical sclerosis	2.01	2.61	0.61	8.94	<0.001	<0.001	0.64 [0.64, 0.64]	0.76 [0.76, 0.76]
Wrist / Chopart	Indeterminate intraosseous lesions	2.28	2.44	0.16	2.41	0.016	0.016	0.48 [0.32, 0.64]	0.61 [0.61, 0.61]
Wrist / Chopart	Osteophytes	2.15	2.57	0.42	6.03	<0.001	<0.001	0.62 [0.62, 0.62]	0.58 [0.58, 0.58]
Wrist / Chopart	Joint space narrowing	2.12	2.57	0.44	6.69	<0.001	<0.001	0.55 [0.46, 0.65]	0.44 [0.23, 0.64]
Wrist / Chopart	Fracture	2.16	2.36	0.20	3.36	<0.001	0.005	0.36 [-1.00, 1.00]	0.52 [0.52, 0.52]
Wrist / Chopart	Indeterminate extraosseous lesions	2.18	2.33	0.15	2.68	0.007	0.015	0.45 [-1.00, 1.00]	0.57 [0.57, 0.57]
CMC / Lisfranc	Subcortical sclerosis	1.99	2.35	0.36	5.28	<0.001	<0.001	0.51 [-0.09, 1.00]	0.73 [0.73, 0.73]
CMC / Lisfranc	Indeterminate intraosseous lesions	2.18	2.43	0.25	3.45	<0.001	0.004	0.67 [0.67, 0.67]	0.61 [0.62, 0.62]

CMC / Lisfranc	Osteophytes	2.06	2.48	0.42	5.05	<0.001	<0.001	0.63 [0.63, 0.63]	0.70 [0.70, 0.70]
CMC / Lisfranc	Joint space narrowing	1.83	2.62	0.80	11.38	<0.001	<0.001	0.74 [0.74, 0.74]	0.62 [0.62, 0.62]
CMC / Lisfranc	Fracture	2.11	2.31	0.20	3.77	<0.001	0.002	0.57 [-1.00, 1.00]	0.63 [0.63, 0.63]
CMC / Lisfranc	Indeterminate extraosseous lesions	2.12	2.31	0.19	3.67	<0.001	0.002	0.77 [0.77, 0.77]	0.63 [0.63, 0.63]
CMC I / MTP I	Subcortical sclerosis	2.05	2.77	0.72	10.85	<0.001	<0.001	0.63 [0.63, 0.63]	0.44 [-0.50, 1.00]
CMC I / MTP I	Indeterminate intraosseous lesions	2.04	2.51	0.47	6.22	<0.001	<0.001	0.62 [0.62, 0.62]	0.71 [0.71, 0.71]
CMC I / MTP I	Osteophytes	2.01	2.57	0.56	7.26	<0.001	<0.001	0.64 [0.64, 0.64]	0.64 [0.64, 0.64]
CMC I / MTP I	Joint space narrowing	2.07	2.46	0.39	6.08	<0.001	<0.001	0.43 [-0.22, 1.00]	0.65 [0.65, 0.65]
CMC I / MTP I	Fracture	2.11	2.36	0.25	4.70	<0.001	<0.001	0.85 [0.85, 0.85]	0.63 [0.63, 0.63]
CMC I / MTP I	Indeterminate extraosseous lesions	2.04	2.36	0.32	6.10	<0.001	<0.001	0.75 [0.75, 0.75]	0.65 [0.65, 0.65]
MCP / MTP II-V	Subcortical sclerosis	1.75	2.37	0.61	7.51	<0.001	<0.001	0.78 [0.78, 0.78]	0.74 [0.74, 0.74]
MCP / MTP II-V	Indeterminate intraosseous lesions	2.20	2.41	0.20	2.87	0.004	0.012	0.70 [0.70, 0.70]	0.70 [0.70, 0.70]
MCP / MTP II-V	Osteophytes	2.03	2.34	0.30	4.50	<0.001	<0.001	0.57 [0.57, 0.57]	0.69 [0.69, 0.69]
MCP / MTP II-V	Joint space narrowing	2.00	2.42	0.42	6.27	<0.001	<0.001	0.63 [0.63, 0.63]	0.66 [0.66, 0.66]
MCP / MTP II-V	Fracture	2.11	2.34	0.23	4.49	<0.001	<0.001	0.91 [0.91, 0.91]	0.61 [0.61, 0.61]
MCP / MTP II-V	Indeterminate extraosseous lesions	2.05	2.33	0.28	5.61	<0.001	<0.001	0.79 [0.79, 0.79]	0.57 [0.57, 0.57]
IP	Subcortical sclerosis	1.84	2.26	0.42	5.55	<0.001	<0.001	0.63 [0.63, 0.63]	0.62 [0.62, 0.62]
IP	Indeterminate intraosseous lesions	2.03	2.30	0.27	3.64	<0.001	0.002	0.61 [0.61, 0.61]	0.55 [0.55, 0.55]
IP	Osteophytes	1.86	2.39	0.53	6.91	<0.001	<0.001	0.62 [0.62, 0.62]	0.65 [0.65, 0.65]

IP	Joint space narrowing	1.61	2.37	0.75	9.02	<0.001	<0.001	0.67 [0.67, 0.67]	0.61 [0.61, 0.61]
IP	Fracture	2.02	2.21	0.19	3.14	0.002	0.008	0.94 [0.94, 0.94]	0.69 [0.69, 0.69]
IP	Indeterminate extraosseous lesions	1.98	2.17	0.20	3.08	0.002	0.008	0.81 [0.81, 0.81]	0.62 [0.62, 0.62]
